# A large prospective investigation of outdoor light at night and obesity in the NIH-AARP Diet and Health Study

**DOI:** 10.1186/s12940-020-00628-4

**Published:** 2020-07-01

**Authors:** Dong Zhang, Rena R. Jones, Tiffany M. Powell-Wiley, Peng Jia, Peter James, Qian Xiao

**Affiliations:** 1 Department of Health and Human Physiology, University of Iowa, E125 Field House, 225 S Grand Ave, Iowa City, Iowa, 52240 USA; 2 Occupational and Environmental Epidemiology Branch, Division of Cancer Epidemiology & Genetics, National Cancer Institute, Rockville, MD USA; 3 National Heart, Lung, and Blood Institute, National Institute on Minority Health and Health Disparities, Bethesda, MD USA; 4 Department of Land Surveying and Geo-Informatics, The Hong Kong Polytechnic University, Hong Kong, China; 5 International Initiative on Spatial Lifecourse Epidemiology (ISLE), Hong Kong, China; 6 Division of Chronic Disease Research Across the Lifecourse (CoRAL), Department of Population Medicine, Harvard Medical School and Harvard Pilgrim Health Care Institute and Department of Environmental Health, Harvard TH Chan School of Public Health, Boston, MA USA; 7 Department of Epidemiology, Human Genetics and Environmental Sciences, The University of Texas Health Science Center at Houston School of Public Health, Houston, TX USA

**Keywords:** Light at night, Light pollution, Obesity, Circadian rhythms

## Abstract

**Background:**

Research has suggested that artificial light at night (LAN) may disrupt circadian rhythms, sleep, and contribute to the development of obesity. However, almost all previous studies are cross-sectional, thus, there is a need for prospective investigations of the association between LAN and obesity risk. The goal of our current study was to examine the association between baseline LAN and the development of obesity over follow-up in a large cohort of American adults.

**Methods:**

The study included a sample of 239,781 men and women (aged 50–71) from the NIH-AARP Diet and Health Study who were not obese at baseline (1995–1996). We used multiple logistic regression to examine whether LAN at baseline was associated with the odds of developing obesity at follow-up (2004–2006). Outdoor LAN exposure was estimated from satellite imagery and obesity was measured based on self-reported weight and height.

**Results:**

We found that higher outdoor LAN at baseline was associated with higher odds of developing obesity over 10 years. Compared with the lowest quintile of LAN, the highest quintile was associated with 12% and 19% higher odds of developing obesity at follow-up in men (OR (95% CI) = 1.12 (1.00, 1.250)) and women (1.19 (1.04, 1.36)), respectively.

**Conclusions:**

Our findings suggest that high LAN exposure could predict a higher risk of developing obesity in middle-to-older aged American adults.

## Background

Obesity has been a growing epidemic in the United States (US), with its prevalence in adults almost tripling between the 1960s and 2018 [1–3]. Although the prevalence of obesity has been relatively stable in the past decade, it remained high and hence the identification of modifiable risk factors for obesity continues to be a priority of public health research [4, 5].

Numerous studies have focused on individual-level risk factors for obesity, such as diet, physical activity, sleep, and psychosocial stress [6–10]. A growing body of literature also suggests that contextual factors, including the built environment, [11–14] food environment, [15–18] neighborhood socioeconomic status, [19–21] neighborhood social support, [22] and neighborhood safety, [23, 24] may play an indispensable role in the development of obesity. Recently, artificial light at night (LAN) has been identified as an environmental factor that may also contribute to obesity [25]. For example, earlier studies have linked shift work with obesity, and it has been postulated that the association was partially driven by LAN [26]. The exposure to LAN can suppress melatonin secretion at night, disrupt circadian rhythms, and alter diurnal behaviors such as sleep and eating, which may in turn lead to the development of obesity [27, 28]. Indeed, some epidemiologic studies have found that higher levels of photometer-measured or self-reported exposure to LAN were positively associated with obesity among women in the UK and US and among older adults in Japan [29–31]. In addition, several studies reported a positive relationship between outdoor LAN, measured by satellite imagery, and obesity [32–34]. Overall, findings from previous studies are intriguing and support a possible role of LAN exposure in obesity development. However, almost all previous studies are cross-sectional and there is a need to examine a prospective association between outdoor LAN and obesity risk in longitudinal studies.

Using data from the NIH-AARP Diet and Health study, a large US cohort of middle- to older-aged men and women, we examined outdoor residential LAN at baseline in relation to the odds of developing obesity over about 10 years. We hypothesized that a higher level of LAN exposure at baseline was associated with higher odds of developing obesity after 10 years. We also conducted subgroup analyses to examine whether the association between LAN exposure and odds of developing obesity may differ by multiple individual and environmental factors including race, education, sleep duration, and neighborhood poverty.

## Methods

### Study sample

We used data from the NIH-AARP Diet and Health Study, which recruited AARP members aged 50–71 from six US states (California, Florida, Louisiana, New Jersey, North Carolina, and Pennsylvania) and two metropolitan areas (Atlanta in Georgia and Detroit in Michigan). Details of the study have been described [35]. Briefly, in 1995–1996, participants completed a baseline questionnaire in which they reported their residential address and a wide range of information on demographics, weight, height, lifestyle, and medical history. In 2004–2006, the same participants completed a follow-up questionnaire that collected the updated information on many of the baseline variables including weight and height. The study was approved by the National Cancer Institute Special Studies Institutional Review Board.

Of the 566,398 participants who completed the baseline questionnaire, 318,713 also completed the follow-up questionnaire. Of these, we excluded 52,210 participants who had missing values on weight and height measures at either baseline or follow-up. We further excluded 2324 participants who reported extreme body mass index (BMI) values (< 15 or > 50 kg/m^2^) or a history of emphysema or end-stage renal disease (*N* = 5071), which may have significant impact on weight changes. We then restricted our analytic subset to “well-geocoded” addresses (i.e., matched to an exact street address or point address only), excluding only street name matches or addresses identified only to the ZIP code or administrative unit level (*N* = 19,327). Our final analytic sample size was 239,781 (142,468 men and 97,313 women). For our main analyses that examined the association between baseline LAN and the odds of prospectively developing obesity at follow-up, we focused on 190,204 participants (114,305 men and 75,899 women) who were not obese (BMI < 30) at baseline.

### Assessment of obesity

Participants reported their height (in inches and feet) and weight (in pounds) at baseline and follow-up, from which BMI (kg/m^2^) was calculated. Obesity was defined as BMI ≥30. We also calculated changes in weight by subtracting baseline weight from weight at follow-up.

### Outdoor LAN measurement

Annual residential outdoor LAN was derived from satellite data from the US Defense Meteorological Satellite Program’s (DMSP’s) Operational Linescan System, maintained by the National Oceanic and Atmospheric Administration’s (NOAA’s) Earth Observation Group. Since previous studies have shown that the low-dynamic range 6-bit DMSP data do not provide sufficient variability in urban areas with high LAN levels, [36] we used the DMSP Global Radiance Calibrated Nighttime Lights high-dynamic range data, which were georectified to a 30 arc-second grid (equivalent to approximately one square kilometer) and transformed into units of radiance (nW/cm^2^/sr) [37]. Exposure estimates were calibrated based on an interannual calibration coefficient provided by NOAA, to ensure comparability across years and satellites. To measure baseline outdoor residential LAN exposure, a nighttime radiance value from 1996 was assigned and linked with residential addresses using ArcGIS (ESRI, Redlands, CA). The LAN variable was then divided into quintiles, with the lowest quintile (referred to as *low LAN exposure*) as the reference group for analyses.

### Statistical analysis

To examine the associations between residential outdoor LAN and odds of developing obesity, we used multiple logistic regression models to calculate odds ratios (OR) and 95% confidence intervals (CI). We used multiple linear regression to calculate beta coefficient and 95% CI for changes in weight as a continuous variable. We considered a series of regression models. In Model 1 (base model), we adjusted for age (continuous), sex (male, female) and baseline BMI (continuous). In Model 2, considered as the main model, we adjusted for some additional potential confounders, including race/ethnicity (non-Hispanic white, non-Hispanic black, Hispanic, Asian or Pacific Islander or American Indian/Alaskan Native, other), education (< 12 years, 12 years, post high school, some college, college/post graduate, unknown), location (six states: California, Florida, Pennsylvania, New Jersey, North Carolina, Louisiana; two metropolitan areas: Atlanta, Georgia, and Detroit, Michigan), marital status (yes, no), and census tract median home value (quintiles), poverty rate (quintiles), and population density (quintiles). We considered important risk factors of obesity as potential covariates, including physical activity, sedentary behavior and diet. However, because LAN influences circadian rhythms that play a central role in regulating diurnal behaviors such as eating and exercise, these behavioral factors are more likely to be mediators, instead of confounders of our analysis. In addition, adjusting them in the model had minimal impact on our results (changes in effect estimates< 1%). Therefore, we decided to not include them in the models. We conducted analyses in the overall population and in men and women separately to examine sex-specific associations. Additional subgroup analyses were performed according to potential effect modifiers, including sleep duration, education, race, and poverty rate at census tract level. To test for trend, we modeled the midpoint of LAN quintiles as continuous variables and used the Wald test to evaluate statistical significance. Statistical significance for interactions was tested using a likelihood ratio test comparing a model with the cross-product term to one without.

## Results

Individual characteristics at baseline (*N* = 239,781) across quintiles of residential outdoor LAN at 1996 were presented in Table 1. Compared with those in lower LAN quintiles, participants in higher LAN quintiles were more likely to be women (46.2% for Q_5_ vs 37.9% for Q_1_) but less likely to be white (84.5% vs 96.3%) or married (59.4% vs 78.2%). In addition, those living in areas with higher LAN were less likely to be current smokers (10.7% vs 9.2%), less likely to report engaging in physical activities ≥5 times/week (18.2% vs 21.6%), or getting7–8 h of sleep per night (40.1% vs 46.9%). Finally, areas with higher levels of LAN also had higher home values (198 k vs 154 k USD) and higher population density (3685 vs 304 per km^2^).

**Table 1:** Baseline Study Characteristics According to LAN at 1996 among 239,781 participants in the National Institutes of Health-AARP Diet and Health Study

	**LAN at 1996**				
**Characteristics**	**Q1**	**Q2**	**Q3**	**Q4**	**Q5**
LAN, nW/cm^2^/sr, Median (Range)	5.3 (0.7, 9.6)	14.2 (9.7, 20.2)	27.2 (20.3, 35.1)	44.6 (35.2, 57.0)	73.8 (57.1, 220.7)
Age at baseline, year, mean (SD)	62.0 (5.3)	61.9 (5.3)	61.9 (5.3)	62.0 (5.3)	61.8 (5.4)
BMI, kg/m^2^, mean (SD)	27.0 (4.4)	27.0 (4.5)	26.8 (4.4)	26.9 (4.6)	27.0 (4.8)
Female, %	37.9	38.2	39.0	41.7	46.2
White, non-Hispanic, %	96.3	95.0	94.0	92.8	84.5
College and post-college, %	38.0	44.2	48.0	47.0	41.5
Married, %	78.2	74.4	71.9	68.3	59.4
Obesity (at baseline), %	20.5	20.5	19.8	20.4	22.3
Smoking, %					
Current	9.2	8.9	9.0	9.1	10.7
Never	38.4	37.9	38.3	38.4	37.9
Physical activity ≥5 times/week, %	21.6	20.8	20.3	19.6	18.2
TV viewing ≤2 h/day, %	35.8	36.6	38.7	38.3	36.1
Nighttime sleep 7–8 h, %	46.9	45.0	44.2	43.3	40.1
Alcohol consumption, g/day, mean (SD)	13.1 (35.4)	12.9 (32.9)	13.0 (32.7)	12.7 (33.5)	12.0 (35.6)
Total energy, kcal/d, mean (SD)	1883 (886)	1849 (832)	1830 (839)	1811 (867)	1826 (944)
HEI-2005 score, mean (SD)	66.9 (11.1)	67.3 (11.0)	67.6 (11.0)	67.8 (11.1)	67.5 (11.3)
Self-reported health, excellent, %	17.9	19.4	20.2	19.6	17.8
Chronic conditions, %					
Heart disease	12.6	12.2	11.8	11.7	11.3
Stroke	1.6	1.5	1.4	1.5	1.4
Cancer	7.6	7.7	8.0	8.0	7.6
Diabetes	7.1	7.2	7.0	7.0	7.8
Census tract median home value, 1kUSD, mean (SD)	154 (121)	190 (157)	214 (159)	213 (149)	198 (145)
Census tract poverty rate, percentage, mean (SD)	8.5 (6.0)	6.9 (5.8)	6.6 (5.9)	7.1 (6.1)	10.3 (8.0)
Census tract population density, per km^2^, mean (SD)	304 (428)	898 (740)	1416 (1079)	1999 (1392)	3685 (3222)

*Abbreviations*: *IQR* interquartile range, *HEI* healthy eating index, *LAN* light at night, *SD* standard deviation

First, we examined the cross-sectional relationship between residential outdoor LAN and obesity at baseline (Table S1). After adjustment for confounders, no association was found between LAN and obesity at baseline in the overall population (*p-for-trend* = *0.50*), or in sex-specific analysis (*p-for-trend* = *0.23* and *0.07* for men and women, respectively).

**Fig. 1:**
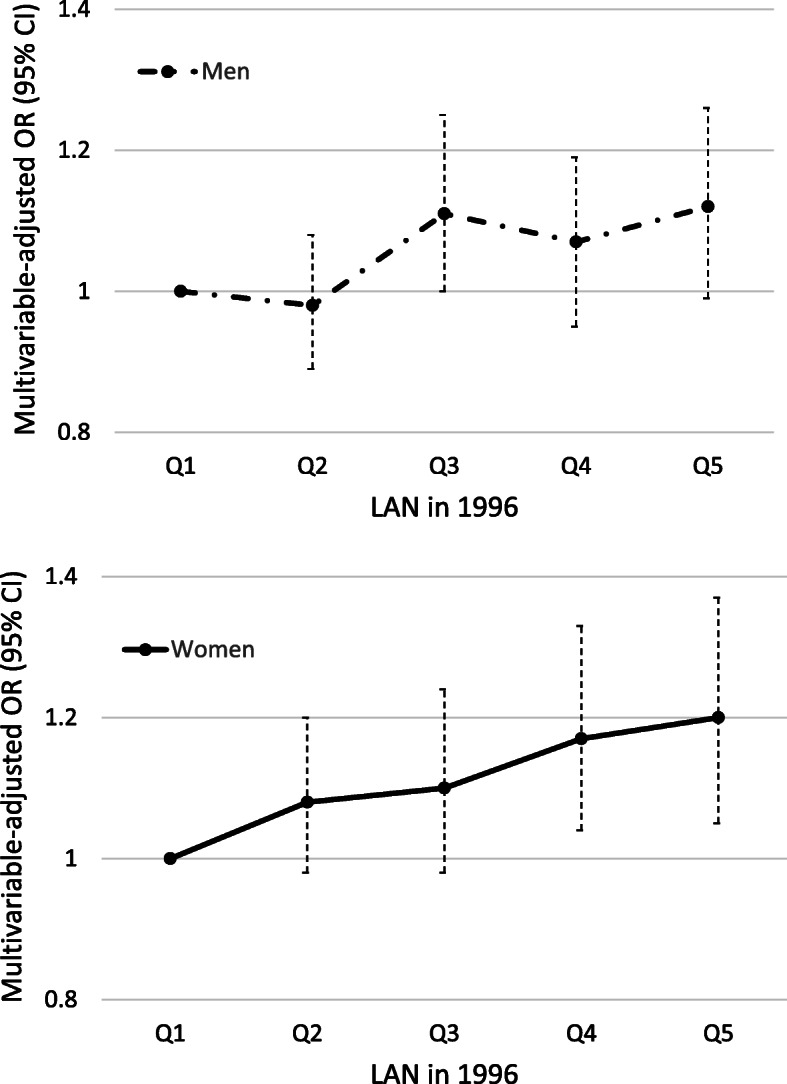
Multivariable-adjusted OR for risk of developing obesity at follow-up. Multivariable-adjusted OR and 95% CI for risk of developing obesity at follow-up among non-obesity participants at baseline according to quintiles of baseline LAN exposure. Vertical lines represent the 95% CIs. Models were adjusted for age, and baseline BMI, race/ethnicity, education, baseline state, marital status, home value, poverty rate, and population density. Abbreviations: CI, confidence interval; LAN, light at night; OR, odds ratio

In prospective analyses, we focused on participants who were not obese at baseline (*N* = 190,204) and found that people living in areas with higher baseline LAN were more likely to develop obesity at follow-up, and the association remained after adjusting for multiple confounders (Table 2). When compared with the lowest quintile of LAN, the highest quintile was associated with 15% higher odds of developing obesity at follow-up (OR_Q5 vs Q1_ (95% CI), 1.15 (1.05–1.25), *p-for-trend* = *0.0001*). In sex-specific analyses, we observed similar results for both men (1.12 (1.00–1.25), *0.039*) and women (1.19 (1.04, 1.36), *0.012;* Table 2 and Fig. 1). We also examined the relationship between baseline LAN exposure and continuous weight change as the outcome (**Table S****2**), and found a significant trend between higher LAN and larger weight gain both in the overall population and those who were not obese at baseline (*p-trend, 0.001* and *0.0001*, respectively).

**Table 2:** Prospective relationship between LAN at baseline and risk of developing obesity at follow-up among non-obesity participants at baseline (*N* = 190,204)

**Obesity at Follow-up**	**LAN in 1996**					***p-for-trend***
	**Q1**	**Q2**	**Q3**	**Q4**	**Q5**	
LAN, nW/cm^2^/sr (Median, Range)	5.3 (0.7, 9.6)	14.2 (9.7, 20.2)	27.2 (20.3, 35.1)	44.6 (35.2, 57.0)	73.8 (57.1, 220.7)	
**Overall**						
No. (%)	3086 (8.3)	3109 (8.1)	3211 (8.1)	3128 (8.1)	3163 (8.8)	
OR (95%CI)						
Model 1	ref	0.98 (0.93, 1.04)	1.02 (0.97, 1.08)	1.02 (0.96, 1.08)	1.08 (1.02, 1.15)	*0.001*
Model 2	ref	1.02 (0.96, 1.10)	1.11 (1.03, 1.19)	1.11 (1.03, 1.20)	1.15 (1.05, 1.25)	*0.001*
**Men (*N*** **= 114,305)**						
No. (%)	1798 (7.7)	1806 (7.5)	1887 (7.8)	1696 (7.4)	1601 (8.1)	
OR (95%CI)						
Model 1	ref	0.95 (0.88, 1.02)	1.04 (0.96, 1.12)	1.00 (0.93, 1.08)	1.12 (1.03, 1.21)	*< 0.001*
Model 2	ref	0.98 (0.90, 1.07)	1.11 (1.01, 1.22)	1.07 (0.96, 1.18)	1.12 (1.00, 1.25)	*0.04*
**Women (*N*** **= 75,899)**						
No. (%)	1288 (9.2)	1303 (9.0)	1324 (8.7)	1432 (9.0)	1562 (9.6)	
OR (95%CI)						
Model 1	ref	1.03 (0.94, 1.13)	1.00 (0.91, 1.09)	1.04 (0.96, 1.14)	1.05 (0.96, 1.15)	*0.24*
Model 2	ref	1.08 (0.97, 1.20)	1.10 (0.98, 1.23)	1.17 (1.04, 1.33)	1.19 (1.04, 1.36)	*0.01*

Model 1: adjusted for age, sex (for overall analysis alone), and baseline BMI

Model 2: adjusted for variables in model 1, and race/ethnicity, education, baseline state, marital status, census tract median home value, poverty rate and population density

*Abbreviations*: *CI* confidence interval, *LAN* light at night, *OR* odds ratio

In subgroup analyses by sleep duration, education, race, and poverty rate at census tract, we found that a positive association between baseline LAN and the odds of developing obesity was generally consistent across subgroups, and none of the *p*-values for interaction were statistically significant (Table S2 for women and Table S3 for men). However, among men, the association between LAN and obesity was found with most of the groups, except for among those reporting less than 12 years of education (0.83 (0.49, 1.40), 0.47) and 9h hours of sleep (0.75 (0.33, 1.69), 0.56) (Table S3), which suggested that the association may differ in men by education status and sleep duration.

## Discussion

In this prospective analysis using data from a large US cohort, we found that individuals living in areas with higher baseline outdoor LAN had higher odds of developing obesity among both men and women. Our findings provide evidence supporting a role of LAN in obesity risk in middle-to-older aged American adults.

Previous cross-sectional studies have consistently reported a positive association between indoor and outdoor LAN and obesity. A Japanese study of 528 elderly individuals found that LAN > 3 lx measured by photometer at home was associated with higher odds of obesity (OR, 1.89; *p-value, 0.02*), abdominal obesity (1.62, *0.04*) and dyslipidemia 1.72, *0.02*) [29]. Similar results were observed in an analysis of the Breakthrough Generations Study using self-reported data about indoor LAN from over 100,000 women aged 16 years or older in the United Kingdom. Findings suggested that women who reported sleeping in darker rooms at night had lower BMI, waist-hip ratio, waist-height ratio and waist circumference when compared to those who reported sleeping in brighter rooms [30]. Two more recent studies analyzed satellite imagery of outdoor LAN as a proxy measure of LAN exposure in relation to obesity and reported similar associations: In one ecological analysis linking outdoor LAN with World Health Organization’s country-level data, Rybnikova et al. reported a positive relationship between LAN and prevalence of overweight and obesity [32]. In another analysis of 8526 adults aged 39–70 in the Korea Genome and Epidemiology Study (KoGES), results also revealed a positive association between outdoor LAN and obesity when comparing high vs low LAN groups (1.20, 95% CI:1.06, 1.36)) [33]. In contrast, we did not observe a significant cross-sectional association between baseline LAN and obesity in our study. Differences in LAN measurement and population samples may partially account for these differences. Home assessments and questionnaire data may better reflect indoor LAN experienced by individuals, which may differ from outdoor LAN measured by satellite imagery. Moreover, the KoGES study, in which the methodology of LAN assessment was most comparable to ours, used the low-dynamic range data of DMSP and used median split to categorize exposure levels. It was also unclear how outdoor LAN levels in KoGES compare with our study sample. Because cross-sectional studies have a limited ability to clarify temporal relationships, it is important to examine the relationship between LAN and obesity in prospective cohorts.

To the best of our knowledge, few studies have examined the prospective relationship between LAN and obesity. In more than 700 elderly Japanese with photometer-measured LAN, Obayashi et al. reported that exposure to an average indoor LAN of 3 lx or higher was associated with higher gain in BMI and waist-to-height ratio over 21 months [31]. Using questionnaire data from 43,722 women in the Sister Study cohort, Park et al. reported that when compared with women who reported sleeping with no light in the room, those who reported sleeping with a television or light on in the room had a higher risk of developing obesity (RR, 1.33; 95% CI, 1.13–1.57; *P* < .001 for trend) [28]. Consistent with these studies, we also found a significant relationship between higher outdoor LAN and higher odds of developing obesity in both men and women over 10 years of follow-up. Taken together, all three prospective studies support a potential role of LAN in obesity risk in adults.

Several possible mechanisms may explain the associations found in our study. Exposure to LAN suppresses melatonin, a key hormone in circadian regulation, and may lead to circadian disruption [38, 39]. A growing body of literature suggests that circadian rhythms play a central role in orchestrating human metabolism and circadian disruption may lead to metabolic disorders including obesity [40, 41]. For example, lab-induced circadian misalignment in human subjects was shown to affect leptin, insulin, glucose and cortisol levels, and the alterations were consistent with metabolic disturbances that may lead to weight gain [42]. In addition, LAN may also promote night-time snacking and disrupt sleep patterns, which may contribute to obesity and metabolic dysfunction. Both animal and human studies have shown that exposure to LAN changes regular timing of food intake, which in turn leads to excess weight gain and obesity [43, 44].

In the last several decades, legislation has been passed in various places to control nighttime light pollution in the US. According to the National Conference of State Legislatures, at least 18 states and territories, including the District of Columbia and Puerto Rico, have laws in place to reduce light pollution [45]. Specific requirements include the installation of shielded light fixtures, using low-glare or low-wattage lighting and following lighting ordinance guidelines. However, light pollution laws are uncommon in the U.S. and a federal regulatory framework for light pollution is missing [46]. It would be informative for future studies to evaluate the effectiveness of light pollution legislation and other interventions to reduce LAN exposure at night in improving population health.

This study has some limitations. First of all, outdoor LAN measured by satellite imagery is only a proxy measure of the actual LAN exposure experienced at the individual level, and we did not have information about indoor LAN and use of curtains and/or blinds, which may lead to exposure misclassification. More information on individuals’ residential history, collected from multiple sources or by other spatial approaches, holds potential to improve the accuracy of this study [47]. Second, there might be other urban environmental factors (e.g., air pollution and noise) that tend to correlate with LAN and may have an impact on obesity [48, 49]. Unfortunately, we were not able to control for these factors due to lack of information in our study, and therefore residual confounding is possible. Third, the advanced sensor technologies could further enrich our cohort datasets by providing multi-temporal, large-coverage images for those variables. Besides, the rapid growth of spatial technologies is expected to provide better-quality, finer-level, and more frequent measurements of LAN in the coming years, [50, 51] which will improve the accuracy of LAN measurement in future studies [47, 52]. Fourth, weight and height were self-reported and are subject to errors that may lead to outcome misclassification. Moreover, we did not have biomarkers to assess metabolic dysfunction in our study population. BMI measures were only available at two time points, which limited our ability to examine longitudinal weight trajectories in relation to LAN. Finally, our sample was predominantly Caucasian and of high socioeconomic status, therefore the findings may not be generalizable to other populations without caution. We did not find evidence supporting a positive association between LAN and obesity in black men and men with lower education. These results suggested potential heterogeneity in the LAN-obesity relationship across racial/ethnic groups and socioeconomic backgrounds. Future studies are needed to further research in those directions.

Despite these limitations, our study has a number of strengths. Most importantly, its longitudinal design has allowed us to examine prospective associations between LAN and the odds of developing obesity. In addition, our large sample size allowed us to conduct subgroup analysis to evaluate the consistency of the relationship according to several sociodemographic, lifestyle, and neighborhood factors.

## Conclusion

In conclusion, our prospective analyses of a large US cohort of older men and women suggest that LAN may contribute to the development of obesity. Our findings could provide potentially important evidence for not only researchers, but also policy makers and public health professionals to develop regulations and interventions that aim at reducing nighttime light pollution in the neighborhood.

## Supplementary information

**Additional file 1: Table S1.** Cross-sectional Relationship between LAN and odds of obesity at baseline (*N* = 239,781). **Table S2.** The association between LAN at baseline and changes in weight between baseline and follow up. **Table S3.** Prospective association ^a^ between baseline LAN and risk of developing obesity at follow-up among non-obese women (*N* = 75,899) at baseline according to subgroups with different education, race, and neighborhood characteristics. **Table S4.** Prospective association ^a^ between baseline LAN and risk of developing obesity at follow-up among non-obese men (*N* = 114,305) at baseline according to different education, race, and neighborhood characteristics.

## Data Availability

Participant data collected during the study may be available upon appropriate request, after de-identification. Depend on staff and funding, and the nature of the request, data could be shared with researchers who provide a methodologically sound research proposal. Proposals should be directed to liaolm@mail.nih.gov to gain access; data requestors will need to get their proposal approved and sign a data access agreement.
