# Supplementary material for: A large prospective investigation of outdoor light at night and obesity in the NIH-AARP Diet and Health Study
Source: Environ Health. 2020 Jul 1;19:74. doi: 10.1186/s12940-020-00628-4 (PMC7329409; doi:10.1186/s12940-020-00628-4)
Supplement: Supplementary file 1 — Additional file 1: Table S1. Cross-sectional Relationship between LAN and odds of obesity at baseline (N = 239,781). Table S2. The association between LAN at baseline and changes in weight between baseline and follow up. Table S3. Prospective association a between baseline LAN and risk of developing obesity at follow-up among non-obese women (N = 75,899) at baseline according to subgroups with different education, race, and neighborhood characteristics. Table S4. Prospective association a between baseline LAN and risk of developing obesity at follow-up among non-obese men (N = 114,305) at baseline according to different education, race, and neighborhood characteristics. [file 12940_2020_628_MOESM1_ESM.docx]

**Table S1** Cross-sectional Relationship between LAN and odds of obesity at baseline (N=239,781)

|  | **LAN in 1996** | | | | |  |
| --- | --- | --- | --- | --- | --- | --- |
| **Obesity at Baseline** | **Q1** | **Q 2** | **Q 3** | **Q 4** | **Q5** | ***p-for-trend*** |
| LAN, nW/cm^2^/sr (Median, Range) | 4.0 (0.7, 8.2) | 12.4 (8.3, 18.0) | 24.9 (18.1, 32.8) | 42.7 (32.9, 54.9) | 73.0 (55.0, 220,7) |  |
| **Overall** | | | | | | |
| No. (%) | 9630 (20.5) | 9954 (20.5) | 9737 (19.8) | 9951 (20.4) | 10305 (22.3) |  |
| OR (95%CI) |  |  |  |  |  |  |
| Model 1 | ref | 1.00 (0.97, 1.03) | 0.95 (0.92, 0.98) | 0.99 (0.96, 1.02) | 1.10 (1.06, 1.13) | *<0.001* |
| Model 2 | ref | 1.06 (1.02, 1.10) | 1.04 (1.00, 1.08) | 1.05 (1.00, 1.09) | 1.02 (0.97, 1.06) | *0.50* |
| **Men (N=142,468)** | | | | | | |
| No. (%) | 5808 (19.9) | 5930 (19.8) | 5742 (19.2) | 5508 (19.4) | 5175 (20.8) |  |
| OR (95%CI) |  |  |  |  |  |  |
| Model 1 | ref | 0.99 (0.95, 1.03) | 0.95 (0.91, 0.99) | 0.97 (0.93, 1.01) | 1.05 (1.01, 1.10) | *0.007* |
| Model 2 | ref | 1.05 (1.00, 1.10) | 1.04 (0.99, 1.10) | 1.04 (0.98, 1.10) | 1.05 (0.99, 1.12) | *0.23* |
| **Women (N=97,313)** | | | | | | |
| No. (%) | 3822 (21.4) | 4024 (21.7) | 3995 (20.8) | 4443 (21.8) | 5130 (24.0) |  |
| OR (95%CI) |  |  |  |  |  |  |
| Model 1 | ref | 1.01 (0.96, 1.06) | 0.96 (0.91, 1.01) | 1.02 (0.97, 1.07) | 1.15 (1.10, 1.21) | *<0.001* |
| Model 2 | ref | 1.08 (1.02, 1.14) | 1.04 (0.97, 1.10) | 1.07 (1.00, 1.14) | 0.99 (0.92, 1.06) | *0.07* |

Model 1: adjusted for age, sex (for overall analysis alone), and baseline BMI.

Model 2: adjusted for variables in model 1, and race/ethnicity, education, baseline state, marital status, census tract median home value, poverty rate and population density.

Abbreviations: CI, confidence interval; LAN, light at night; OR, odds ratio.

**Table S2** The association between LAN at baseline and changes in weight between baseline and follow up.

|  | **LAN in 1996** | | | | |  |
| --- | --- | --- | --- | --- | --- | --- |
| **β (95%CI), lb** | **Q1** | **Q2** | **Q3** | **Q4** | **Q5** | ***p-for-trend*** |
| **Overall** | | | | | | |
| Model 1 | ref | -0.08 (-0.17, 0.02) | 0.02 (-0.07, 0.12) | 0.07 (-0.02, 0.16) | 0.08 (-0.14, 0.17) | *0.004* |
| Model 2 | ref | -0.03 (-0.13, 0.08) | 0.13 (0.02, 0.25) | 0.20 (0.07, 0.32) | 0.14 (0.01, 0.28) | *0.001* |
| **Nonobese participants at baseline** | | | | | | |
| Model 1 | ref | -0.10 (-0.18, -0.01) | 0.00 (-0.09, 0.09) | 0.01 (-0.08, 0.10) | 0.07 (-0.02, 0.17) | *0.02* |
| Model 2 | ref | -0.03 (-0.13, 0.08) | 0.14 (0.02, 0.25) | 0.16 (0.04, 0.28) | 0.19 (0.06, 0.32) | *0.0001* |

Model 1: adjusted for age, sex, and baseline BMI.

Model 2: adjusted for variables in model 1, and race/ethnicity, education, baseline state, marital status, census tract median home value, poverty rate and population density.

Abbreviations: CI, confidence interval; LAN, light at night.

**Table S3** Prospective association ^a^ between baseline LAN and risk of developing obesity at follow-up among non-obese women (N=75,899) at baseline according to subgroups with different education, race, and neighborhood characteristics.

|  |  | **LAN in 1996** | | | | |  |  |
| --- | --- | --- | --- | --- | --- | --- | --- | --- |
|  | **N** | **Q1** | **Q2** | **Q3** | **Q4** | **Q5** | ***p-value for interaction*** | ***p-for-trend*** |
| Sleep duration, hrs |  |  |  |  |  |  | *0.66* |  |
| <7 | 18,317 | ref | 0.96 (0.78, 1.19) | 1.07 (0.85, 1.33) | 1.16 (0.91, 1.47) | 1.15 (0.89, 1.49) |  | *0.07* |
| 7-8 | 34,005 | ref | 1.10 (0.93, 1.30) | 1.09 (0.91, 1.31) | 1.08 (0.89, 1.31) | 1.15 (0.93, 1.42) |  | *0.30* |
| 9+ | 1,748 | ref | 1.03 (0.52, 2.03) | 1.20 (0.58, 2.48) | 1.08 (0.46, 2.51) | 1.51 (0.63, 3.61) |  | *0.28* |
|  |  |  |  |  |  |  |  |  |
| Education |  |  |  |  |  |  | *0.59* |  |
| Less than 12 years | 2,755 | ref | 1.47 (0.91, 2.37) | 1.14 (0.65, 1.99) | 1.52 (0.86, 2.69) | 2.09 (1.12, 3.89) |  | *0.02* |
| 12 years or more | 71,182 | ref | 1.05 (0.94, 1.17) | 1.08 (0.96, 1.22) | 1.14 (1.01, 1.29) | 1.15 (1.00, 1.31) |  | *0.05* |
|  |  |  |  |  |  |  |  |  |
| Race |  |  |  |  |  |  | *0.52* |  |
| White | 69,814 | ref | 1.07 (0.96, 1.19) | 1.10 (0.97, 1.23) | 1.17 (1.03, 1.33) | 1.19 (1.04, 1.36) |  | *0.01* |
| Black | 2,843 | ref | 1.52 (0.79, 2.91) | 1.05 (0.53, 2.09) | 1.01 (0.49, 2.05) | 1.11 (0.54, 2.26) |  | *0.54* |
|  |  |  |  |  |  |  |  |  |
| Census tract poverty rate ^b^ |  |  |  |  |  |  | *0.46* |  |
| Low | 37,400 | ref | 1.04 (0.89, 1.21) | 1.02 (0.86, 1.20) | 1.18 (0.99, 1.40) | 1.22 (1.00, 1.48) |  | *0.01* |
| High | 38,499 | ref | 1.12 (0.97, 1.29) | 1.17 (1.00, 1.37) | 1.16 (0.98, 1.38) | 1.17 (0.98, 1.40) |  | *0.25* |

^a^ Adjusted for age, baseline BMI, race/ethnicity, education, baseline state, marital status, home value, poverty rate, and population density.

^b^ low and high neighborhood poverty was defined according to median (6.6%).

Abbreviations: CI, confidence interval; LAN, light at night; OR, odds ratio.

**Table S4** Prospective association ^a^ between baseline LAN and risk of developing obesity at follow-up among non-obese men (N=114,305) at baseline according to different education, race, and neighborhood characteristics.

|  |  | **LAN in 1996** | | | | |  |  |  |
| --- | --- | --- | --- | --- | --- | --- | --- | --- | --- |
|  | **N** | **Q1** | **Q2** | **Q3** | **Q4** | **Q5** | ***p-value for interaction*** | | ***p-for-trend*** |
| Sleep duration, hrs |  |  |  |  |  |  | *0.94* | |  |
| <7 | 24,252 | ref | 1.01 (0.84, 1.22) | 1.10 (0.90, 1.35) | 1.05 (0.84, 1.31) | 1.09 (0.86, 1.39) |  | | *0.54* |
| 7-8 | 52,224 | ref | 1.02 (0.89, 1.16) | 1.14 (0.98, 1.32) | 1.07 (0.91, 1.26) | 1.15 (0.96, 1.38) |  | | *0.15* |
| 9+ | 2,482 | ref | 0.69 (0.37, 1.27) | 0.99 (0.51, 1.94) | 0.64 (0.30, 1.34) | 0.75 (0.33, 1.69) |  | | *0.56* |
|  |  |  |  |  |  |  |  | |  |
| Education |  |  |  |  |  |  | *0.55* | |  |
| Less than 12 years | 4,155 | ref | 0.86 (0.58, 1.26) | 0.83 (0.53, 1.30) | 1.03 (0.63, 1.67) | 0.83 (0.49, 1.40) |  | | *0.47* |
| 12 years or more | 107,967 | ref | 0.99 (0.90, 1.08) | 1.13 (1.02, 1.25) | 1.06 (0.96, 1.18) | 1.13 (1.00, 1.27) |  | | *0.03* |
|  |  |  |  |  |  |  |  | |  |
| Race |  |  |  |  |  |  | *0.68* | |  |
| White | 106,992 | ref | 0.97 (0.89, 1.06) | 1.09 (0.99, 1.20) | 1.04 (0.94, 1.16) | 1.09 (0.96, 1.22) |  | | *0.11* |
| Black | 2,265 | ref | 1.05 (0.56, 1.99) | 1.63 (0.81, 3.32) | 1.26 (0.59, 2.69) | 1.29 (0.60, 2.78) |  | | *0.84* |
|  |  |  |  |  |  |  |  | |  |
| Census tract poverty rate ^b^ |  |  |  |  |  |  | *0.25* | |  |
| Low | 65,018 | ref | 1.02 (0.90, 1.15) | 1.10 (0.97, 1.25) | 1.12 (0.98, 1.29) | 1.14 (0.97, 1.34) |  | | *0.08* |
| High | 49,287 | ref | 0.95 (0.84, 1.08) | 1.16 (1.00, 1.34) | 1.00 (0.85, 1.17) | 1.09 (0.92, 1.29) |  | | *0.23* |

^a^ Adjusted for age, baseline BMI, race/ethnicity, education, baseline state, marital status, home value, poverty rate, and population density.

^b^ low and high neighborhood poverty was defined according to median (6.6%).

Abbreviations: CI, confidence interval; LAN, light at night; OR, odds ratio.
